# Influenza A Virus Infection of Intestinal Epithelial Cells Enhances the Adhesion Ability of Crohn’s Disease Associated *Escherichia coli* Strains

**DOI:** 10.1371/journal.pone.0117005

**Published:** 2015-02-23

**Authors:** Marta Aleandri, Maria Pia Conte, Giovanna Simonetti, Simona Panella, Ignacio Celestino, Paola Checconi, Massimiliano Marazzato, Catia Longhi, Paola Goldoni, Mauro Nicoletti, Nicolas Barnich, Anna Teresa Palamara, Serena Schippa, Lucia Nencioni

**Affiliations:** 1 Department of Public Health and Infectious Diseases, “Sapienza” University, Rome, Italy; 2 IRCCS San Raffaele Pisana, Telematic University, Rome, Italy, Rome, Italy; 3 CEINGE-Biotecnologie Avanzate, Naples, Italy; 4 Department of Public Health and Infectious Diseases, Pasteur Institute Cenci Bolognetti Foundation, “Sapienza” University, Rome, Italy; 5 Department of Experimental Sciences and Clinics “G. D’Annunzio” University, Chieti, Italy; 6 Clermont University, M2iSH, UMR 1071 INSERM/University of Auvergne, Clermont-Ferrand, France; INSERM, FRANCE

## Abstract

Modifications of intestinal glycoreceptors expression, in particular CEACAM6, typically found in ileal Crohn's disease (CD), favor, among the commensal species of microbiota, the enrichment in *Escherichia coli*. Removal of protein glycosidic residues by neuraminidase, a sialidase typical of influenza virus, increases adhesion ability of *Escherichia coli* to Caco-2 intestinal cells. In this study we investigated whether influenza virus infection of human intestinal epithelial cells could influence the adhesiveness of different *Escherichia coli* strains isolated from CD patients by altering surface glycoreceptors. Influenza virus infection of intestinal cells increased exposure of galactose and mannose residues on the cell surface. In particular, glycoreceptors Thomsen-Friedenreich and CEACAM6 were over-expressed in influenza virus infected cells. In the same experimental conditions, a significant increase in bacterial adhesiveness was observed, independently of their own adhesive ability. The increase was reverted by treatment with anti-TF and anti-CEACAM6 antibodies. Interestingly, influenza virus was able to efficiently replicate in human primary intestinal cells leading to TF exposure. Finally, intestinal infected cells produced high levels of pro-inflammatory cytokines compared to control. Overall these data suggest that influenza virus infection, could constitute an additional risk factor in CD patients.

## Introduction

Inflammatory bowel diseases (IBD), including Crohn’s disease (CD), are immune-mediated disorders originating from a breakdown of the normal symbiosis between the mucosal immune responses and the commensal flora [1,2]. Several factors can contribute to disease’s pathogenesis such as susceptibility [3], defects in mucosal barrier function [4] and imbalance in the gut microbiota composition [5]. In particular, a compositional shift with depletion in specific types of commensal species and enrichment in harmful bacteria, such as specific genotypes of the mucosa-associated *Escherichia coli* (*E. coli*) species, has been reported in the CD-associated microbiota [6–9]. These strains, called *E. coli* AIEC (adherent/invasive *E. coli*), are characterized by strong adhesive-invasive properties [10–13] and are able to invade and replicate within epithelial cells and macrophages [14,15]. Changes in the mucosal-associated flora may also depend on the abnormal interaction between intestinal cells and microbial lectins caused by an altered glycosylation pattern of mucosal proteins, which is widely observed in CD patients [16]. These alterations include the increased expression of oncofetal glycans such as the Thomsen-Friedenreich (TF) receptor and an abnormal ileal expression of carcinoembryonic antigen-related cell adhesion molecule 6 (CEACAM6), both of which are recognized specifically by *E. coli* adhesins [17–21]. In particular, *E. coli* AIEC strains bind the mannosylated glycoreceptor CEACAM6 by a variant of the FimH, a mannose-specific type 1 pili adhesin [22,23]. In normal epithelium, the TF (Galactoseβ1-3αNAcetylgalactosamine, Galβ1-3αGalNac) structure is concealed by sialic acids (SA) to form branched and complex O-glycans [24]. We previously demonstrated that treatment of intestinal cells with *Clostridium perfringens* neuraminidase, an enzyme characterized by sialidase activity that cuts SA from the Gal residues, caused a significant increase in the adhesive ability of *E. coli* strains isolated from bioptic samples of CD pediatric patients, and suggested that this event could be linked to over-exposure of receptors, such as TF antigen [17]. NA is a glycoprotein normally present on the envelope of all influenza viruses that helps the release of mature viral particles from the host cells, cutting SA residues on the cell surface. Interestingly, influenza virus (IV) infection has been shown to induce over-expression of CEACAM6 protein, probably via interaction with NA followed by activation of the Src/Akt signaling pathway in lung epithelial cells [25].

These findings prompted us to hypothesize that infection of intestinal epithelial cells with IV alters the glycosylation pattern of mucosal proteins and thereby increases bacterial adhesiveness. Several studies provide evidence of the ability of IV to infect the gut epithelium. Shu et al. [26] found that receptors for IV were also abundantly expressed on gastrointestinal (GI) epithelial cells, which are highly permissive for their replication [27,28]. Accordingly, gastrointestinal symptoms such as diarrhea, vomiting, and abdominal pain as well as fecal detection of IV has been reported in seasonal influenza [29–35]. In addition, Okayama et al. [36] reported a case of hemorrhagic colitis after infection with seasonal influenza A H3N2 virus.

Based on these observations we decided to investigate whether the infection of intestinal epithelial cells with influenza A virus favors the adhesive ability of three *E. coli* strains, AIEC LF82, AIEC LF82 Δ*fimH* isogenic mutant and S15, a FimH negative strain isolated from the intestinal mucosa of a CD patient [18]. We found that IV infection caused: i) a progressive increase in TF antigen exposure; ii) a significant increase in mRNA level of CEACAM6 and its expression on the cell surface. These events were directly related to the increased ability of the *E. coli* strains to adhere to intestinal epithelial cells. More interestingly, the clinical isolate S15 as well as AIEC LF82 Δ*fimH*, were able to significantly adhere to epithelial cells, suggesting that other unknown bacterial adhesins and different cellular receptors unmasked during IV infection, could be involved in bacterial adhesion. Moreover IV infection led to secretion of pro-inflammatory cytokines in the supernatants of intestinal epithelial cells.

## Methods

### Cell Cultures

Human colorectal adenocarcinoma Caco-2 cell line (ATCC HTB-37) was cultured in DMEM (Euroclone) supplemented with 10% fetal bovine serum (FBS, Gibco), 2 mM glutamine, 100 U/ml penicillin, 100 mg/ml streptomycin (Euroclone). Cell viability was estimated by Trypan blue (0.02%) exclusion (Sigma-Aldrich).

Primary human intestinal epithelial cells (Clonetics InEpC’s, Lonza) were grown in SmBM smooth muscle cell basal medium added with the supplements and growth factors insulin, hFGF-B, hEGF, FBS and gentamicin/amphotericin-B (SmGM-2 BulletKit, Lonza) at 33°C.

### Virus production, infection and titration

Influenza A/Puerto Rico/8/34 H1N1 virus (PR8 virus) was grown in the allantoic cavities of 10-day-old embryonated chicken eggs. After 48 hrs at 37°C, the allantoic fluid was harvested and centrifuged at 5000 rpm for 30 min to remove cellular debris.

Caco-2 cells plated for 48 hrs were infected with PR8 at different multiplicities of infection (MOI), incubated for 1 hr at 37°C, washed with PBS, and then incubated with medium without antibiotics and supplemented with 2% FBS for different time points.

InEpC plated for 8 days were infected with PR8 at different MOI for 3 hrs at 33°C and then incubated with medium without antibiotics and supplemented with 2% FBS for 24 hrs.

Mock infection was performed with the same dilution of allantoic fluid from uninfected eggs.

Virus production was determined in supernatants of Caco-2 cells measuring haemagglutinin units (HAU) according to standard procedures [37]. Virus titer in InEpC was analyzed by Real Time PCR. Briefly, total RNA was extracted in supernatants of infected cells with Viral Nucleic acid Extraction kit (Geneaid) according to the manufacturer’s protocol. The number of viral RNA M1 copies was determined by quantitative Real Time RT-PCR using both One Step Influenza A/B r-gene and Quanti FluA kits (Argene).

Viral neuraminidase activity was measured on both supernatants of virus infected cells (0.8 MOI, 24 hrs p.i.) and of treated *Clostridium perfringens* neuraminidase type V (Cl NA) (Sigma-Aldrich) cells (2 μg/ml), with NA-Fluor Influenza Neuraminidase assay Kit (Life Technologies). The enzymatic activity was measured after incubation with a fluorescently labeled substrate, methyl-umbelliferyl-N-acetyl neuraminic acid (MUNANA) and expressed as concentration of the end product, the 4-methylumbelliferone (4-MU). Fluorescence was read on a reader with excitation and emission filters of 355 nm and 460 nm respectively.

### Bacterial strains

The prototype adherent/invasive *E. coli* (AIEC) LF82 strain, isolated from a chronic ileal lesion of a Crohn’s disease patient, was a generous gift by Dr. Arlette Darfeuille-Michaud, University of Auvergne, France. The LF82 isogenic mutant deleted of *fimH* gene was generated by PCR as described by Boudeau et al. [38]. *E. coli* S15 was a FimH negative strain isolated from ileum of CD pediatric patient attending the Pediatric Gastroenterology and Liver Unit, Sapienza University of Rome [18]. To obtain maximal fimbrial expression, bacterial colonies were grown overnight in nutrient agar, re-suspended in sterile saline solution and left for 48 hrs at room temperature, as described by Martin et al. [17].

### Co-infection and adhesion assays

Caco-2 cells, seeded in 24-well plates at a density of 1x10^5^ cells/well, were incubated for 48 hrs at 37°C, and then infected with PR8 at different MOI. Twenty-four hrs after viral infection, the number of 4x10^5^ cells/well was counted by Tripan Blue assay (Sigma Aldrich). The monolayers were washed with PBS, and co-infected with 1x10^7^ bacteria/ml (MOI 25) estimated by both the number of colony-forming units (CFU) and optical density. After 2 hrs incubation at 37°C, cells were washed with PBS and lysed with cold 0.1% Triton X-100. Bacterial cells were plated and CFU were determined after 24 hrs. *E. coli* strains were considered adherent when the mean adhesion index was equal or superior to 1 bacterium per cell.

Moreover, intestinal epithelial cell monolayers were plated for 71 hrs, and then treated with Cl NA 2 μg/ml. After 1 hr incubation at 37°C cells, were infected with bacteria (MOI 25) as described above.

To assess the effect of TF and CEACAM6 antibodies (Abs) on bacterial adhesion, cell monolayers, after 48 hrs plating, were infected with PR8 0.8 MOI for 24 hrs. Then cells were incubated with anti-TF (Abcam) or with anti-CEACAM6 Abs (Santa Cruz Biotechnology) for 2 hrs at 4°C. The cells were then washed with PBS and co-infected with 1x10^7^ bacteria/ml as described above.

### Fluorescence microscopy

Immunofluorescence analysis was performed in Caco-2 cells plated for 48 hrs at a density of 1x10^5^ cells/well, then infected with PR8 (0.2 or 0.8 MOI) and incubated for the following 4, 6, and 24 hrs. Cells were fixed with 4% paraformaldehyde (Sigma-Aldrich) in PBS, washed and permeabilized with PBS/Triton X-100 (Sigma-Aldrich). They were then blocked for 45 min in 3% milk diluted in PBS and stained as follows. To assess exposure of galactose or mannose residues, cells were incubated for 1 hr at 37°C with 20 μg/ml lectin *Arachis hypogea* (PNA, gal(1→3)galNac) FITC-conjugated (Sigma-Aldrich) or with 5 μg/ml lectin Concanavalin A (ConA, α-D-mannosyl and α-D-glucosyl) FITC-conjugated (United States Biochemical Corporation); to assess exposure of TF or CEACAM-6, cells were incubated for 45 min with mouse monoclonal anti-TF or mouse monoclonal anti-CEACAM6 Abs and then with goat anti-mouse Alexa-Fluor 488 Abs (Invitrogen).

For HA detection, cells were fixed as described above, blocked for 45 min with 3% goat serum diluted in PBS and incubated for 45 min with mouse anti-HA (Santa Cruz Biotechnology) and secondary goat anti-mouse Alexa-Fluor 568 Abs (Invitrogen).

As a control, Caco-2 mock-infected cells were plated at density of 1x10^5^ cells/well and after 72 hrs plating were incubated with 20 μg/ml of PNA or 5 μg/ml of ConA, both FITC-conjugated, to detect cell glycosylation state. Moreover Caco-2 mock-infected cell were incubated with mouse anti-TF or mouse anti-CEACAM6 Abs to evaluate basal expression of the receptors.

For each immunofluorescence assay, the percentage of positive FITC- or rodhamine-labelled cells *versus* total cells, taken as 100%, was calculated. Cells counts were based on examination of different fields from 3 independent experiments at magnification of 100X.

InEpC were plated for 8 days at a density of 1x10^5^ cells/well, then infected with PR8 at 1.6 MOI and incubated for 24 hrs. Cells were fixed with 4% paraformaldehyde (Sigma-Aldrich) in PBS, washed and blocked for 45 min in 3% milk diluted in PBS and stained with mouse monoclonal anti-TF and then with goat anti-mouse Alexa-Fluor 488 Abs.

### RNA extraction and real-time PCR

Total RNA was isolated using the RNeasy Mini kit (Qiagen) following the manufacturer’s protocol. The quantity and quality of isolated RNA were determined spectrophotometrically (Pearl IMPLEN nanophotometer). An equal amount of the total RNA was used as template to generate cDNA using iScript cDNA Synthesis Kit (Bio-Rad). An aliquot of the cDNA was then subjected to 40 cycles of Real-time PCR amplification (95°C, 10 sec; 60°C, 30 sec) using the iQ SYBR Green Supermix and LightCycler iQ 5 (Bio-Rad). To ensure that the primers produced a single and specific PCR amplification product, a melting curve was performed at the end of each PCR cycle.

Relative quantitative evaluation was performed by the comparative ΔΔCt method. The mean ΔCt obtained in control cells (mock-infection) for CEACAM6 gene [25] (CACAACCTGCCCCAGAATCGTAT forward; TTGGGCAGCTCCGGGTATACATG reverse) was used as calibrator, after normalization to endogenous control β-actin (ACCAACTGGGACGACATGGAGAAA forward; TAGCACAGCCTGGATAGCAACGTA reverse) and GAPDH (GTCGGAGTCAACGGATTT forward; CAACAATATCCACTTTACCAGAG reverse) genes. The results are presented as fold difference relative to control.

### Western Blot Analysis

Mock- and PR8-infected cells were treated with lysis buffer RIPA [20 mM Tris-HCl pH 7.5, 150 mM NaCl, 1 mM Na_2_EDTA, 1mM EGDA, 1% NP-40, 1% sodium deoxycholate, 2.5 mM sodium pyrophosphate, 1 mM 6 β-glycerophospahte, 1% Triton X-100, 0.1% sodium dodecyl sulfate-(SDS)] supplemented with phenylmethylsulfonylfluoride, a phosphatase inhibitor mixture (Sigma-Aldrich). Cell lysates were incubated for 30 min on ice, centrifuged at 13000×*g* for 30 min. Supernatants were collected and assayed to determine their protein concentration (Bradford method, Bio-Rad). Samples were run on SDS-PAGE, blotted onto nitrocellulose membranes, and, after blocking with 10% nonfat dry milk, were stained with anti-CEACAM6 antibody. Secondary Abs were peroxidase-conjugated (Jackson ImmunoResearch). Blots were developed with the ECL-Plus Detection System (GE Healthcare) and subjected to densitometric scanning by QuantityOne program.

### Pro-inflammatory cytokines analysis

Cytokine and Chemokine levels in supernatants of Caco-2 cells and of InEpC were determined by a Bioplex multiplex assay; plates (Millipore) were read on a Bio-Plex MAGIPIX instrument. Data were analyzed using the Bio-pro software and the results were expressed as fold induction to control (= 1). All standards and sample were run in duplicate.

### Statistical analysis

Data were expressed as the mean ± S.D. The statistical significance between different experimental conditions was determined by the paired Student’s t-test (*P* values of <0.05 were considered significant).

## Results

### Influenza virus infection enhances *E. coli* adhesion to Caco-2 cells

To assess the effect of viral infection on the bacterial adhesive ability, experiments of co-infection of intestinal cells with IV and *E. coli* strains were performed. Monolayers of Caco-2 cells (48 hrs post plating) were infected with PR8 virus at different MOI (0.2, 0.4, and 0.8) for 24 hrs and the cells were then infected with *E. coli* AIEC LF82, *E.coli* LF82 Δ*fimH* or *E. coli* S15 strains for 2 hrs. As control, bacterial adhesion of strains was monitored in mock-infected cells treated or not with *Clostridium perfringens* neuraminidase (Cl NA) for 1 hr. As shown in Table 1, a progressive increase in the adhesion level of *E. coli* strains was found in cells pre-infected with PR8. Interestingly, the adhesion indices observed in PR8 pre-infected cells, were higher than those observed in mock-infected cells pre-treated with Cl NA, thus indicating that infection with the whole virus is more effective than treatment with purified Cl NA in promoting bacterial adhesion. Accordingly, NA enzymatic activities measured in the supernatants of virus-infected cells and in the supernatants of treated Cl NA cells showed no significant differences. In fact the concentration of 4-MU (end product) was 1.7 μM and 1.5 μM, respectively. These data suggest that the enhanced adhesion ability of *E. coli* strains on virus-infected cells is not only due to the NA activity.

**Table 1:** Bacterial adhesion of *E. coli* strains increases in influenza virus-infected cells.

	Mock Inf		PR8		
Strain	Ctr	Cl NA	0.2	0.4	0.8
S15	0.95±0.61	2.58±0.74	**2.74±0.73	**4.26±0.94	**5.50±2.70
LF82	6.99±4.42	12.66±2.83	**20.67±7.37	**28.81±11.13	**42.76±12.40
LF82-Δ*fimH*	4.49±2.16	3.03±0.57	5.81±1.43	6.90±1.47	**13.44±4.94

Caco-2 cells were infected with influenza virus (PR8) at different MOI (0.2, 0.4 and 0.8) for 24 hrs. Cells were then infected with *E. coli* LF82, LF82 *ΔfimH* or S15 (MOI 25) for 2 hrs. As controls, cells were mock-infected and treated with neuraminidase of *Clostridium perfringens* (Cl NA) or not (Ctr) before bacterial infection. Cells were then lysed and the number of colony-forming units (CFU) was determined by plating (see Methods). The mean adhesion index values of the three strains are reported. Data represent the mean ± SD of three independent experiments, each performed in triplicate.

**P<0.001 *vs*. Ctr.

### Influenza virus infection enhances galactose and mannose residues exposure

**Fig 1:**
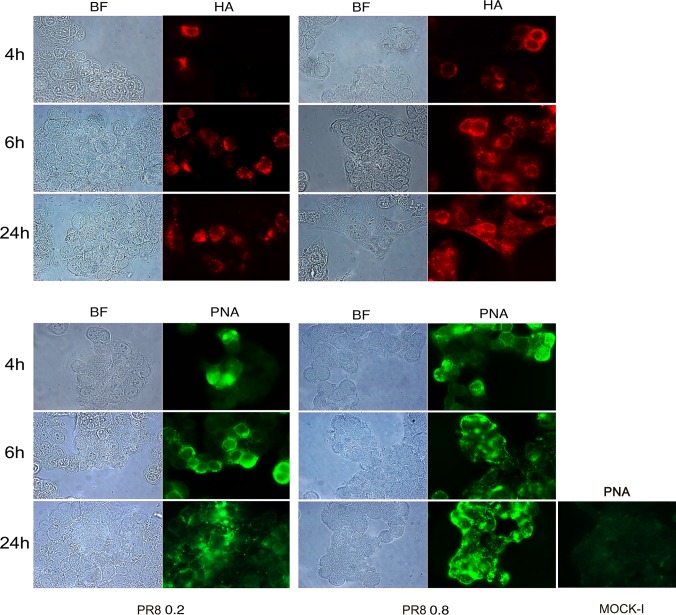
Influenza virus increases exposure of galactose residues in intestinal cells. Caco-2 cells were infected with PR8 at 0.2 and 0.8 MOI and maintained for 4, 6 and 24 hrs at 37°C. Cells were then fixed and samples were incubated with anti-HA Abs (upper panel) to follow the progression of viral infection and with PNA (bottom panel) to detect galactose residues in PR8 infected cell and in the control cell (Mock-I), as described in Methods. BF: Bright field. Results are shown for one representative experiment of the three performed at magnification of 100X.

**Fig 2:**
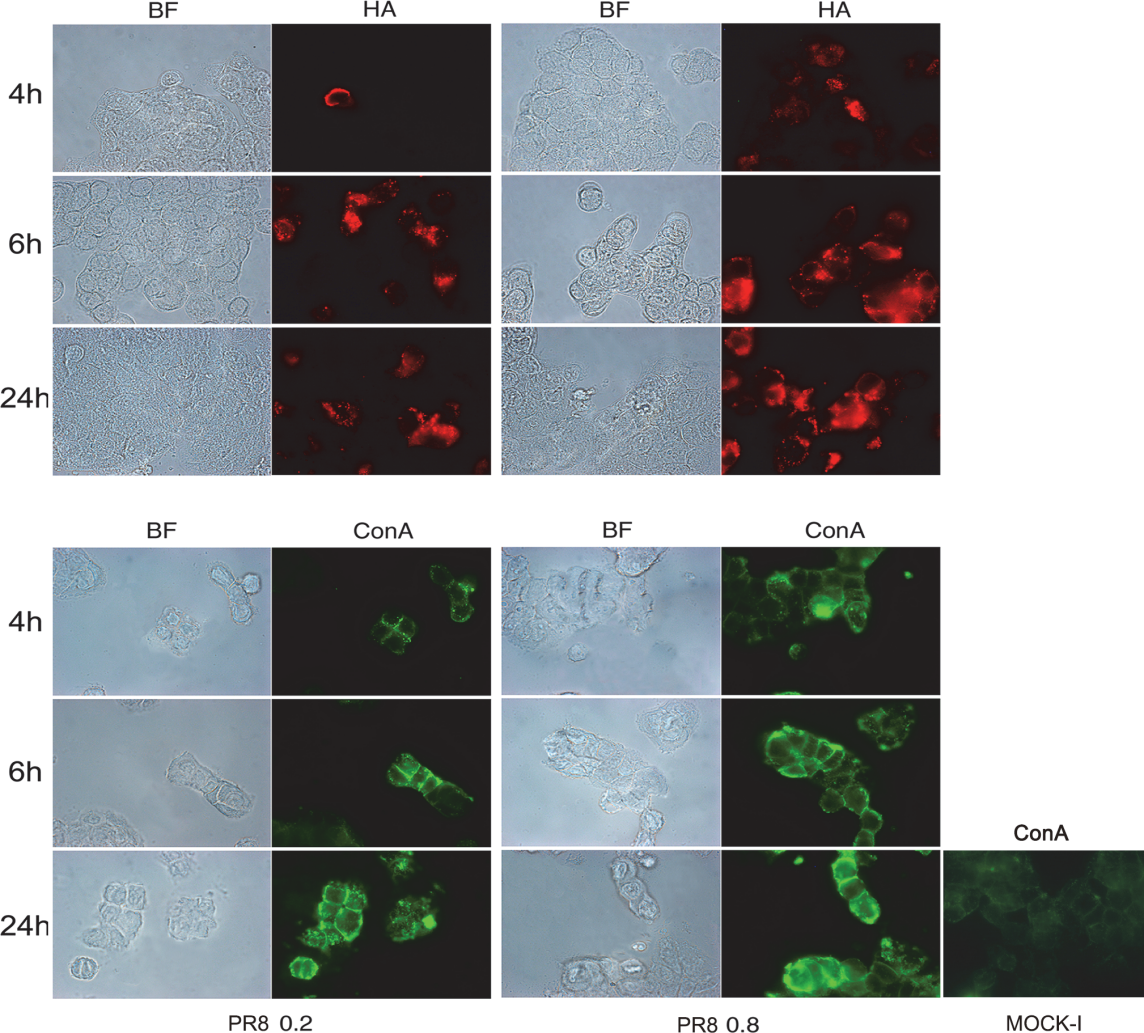
Influenza virus increases exposure of mannose residues in intestinal cells. Caco-2 cells were infected with PR8 at 0.2 and 0.8 MOI and maintained for 4, 6 and 24 hrs at 37°C. Cells were then fixed and samples were incubated with anti-HA Abs (upper panel) to follow the progression of viral infection and with ConA (bottom panel) to detect mannose residues, in PR8 infected cell and in the control cell (Mock-I), as described in Methods. BF: Bright field. Results are shown for one representative experiment of the three performed at magnification of 100X.

Mucosal glycosylation changes have been observed in IBD patients [19,20] and these alterations could promote the interaction between intestinal mucosa and microbial lectins. Thus, to investigate whether the increased bacterial adhesion observed after viral infection was due to some virus-induced glycosylation changes on the intestinal cell surface, we performed immunofluorescence analysis. Cells, at 48 hrs post plating, were infected with PR8 at 0.2 or 0.8 MOI and incubated for the following 4, 6 and 24 hrs. The progression of viral infection (from 4 to 24 hrs post infection, p.i) was followed by labeling infected cells with anti-HA Abs and by counting positive cells with respect to total cells. The percentage of infected cells at 0.2 MOI ranged from 11%±1 to 29%±7 and at 0.8 MOI from 24.4%±4 to 46%±9. Jointly, galactose and mannose residues were detected by labeling cells with FITC-conjugated *Arachis hypogea* lectin (PNA) or FITC-conjugated Concanavalin A lectin (ConA), respectively. Results obtained showed a progressive exposure of galactose (Fig. 1) and mannose (Fig. 2) residues on the cell surface, in a time-dependent manner (at 0.2 MOI the percentage of galactose and mannose positive cells ranged from 6.5%±0.7 to 32%±0 and from 13%±1 to 40%±14 respectively) with maximal exposure at 24 hrs p.i. respect to the control (Mock-I).

These events were more evident in cells infected with PR8 at high MOI (the percentage of galactose and mannose positive cells ranged from 29%±6 to 71%±6 and from 9%±0.6 to 63%±25 respectively), indicating that viral infection was able to induce glycosylation changes on the membrane of infected intestinal cells.

### Influenza virus infection enhances TF antigen exposure and CEACAM6 mRNA and protein expression

**Fig 3:**
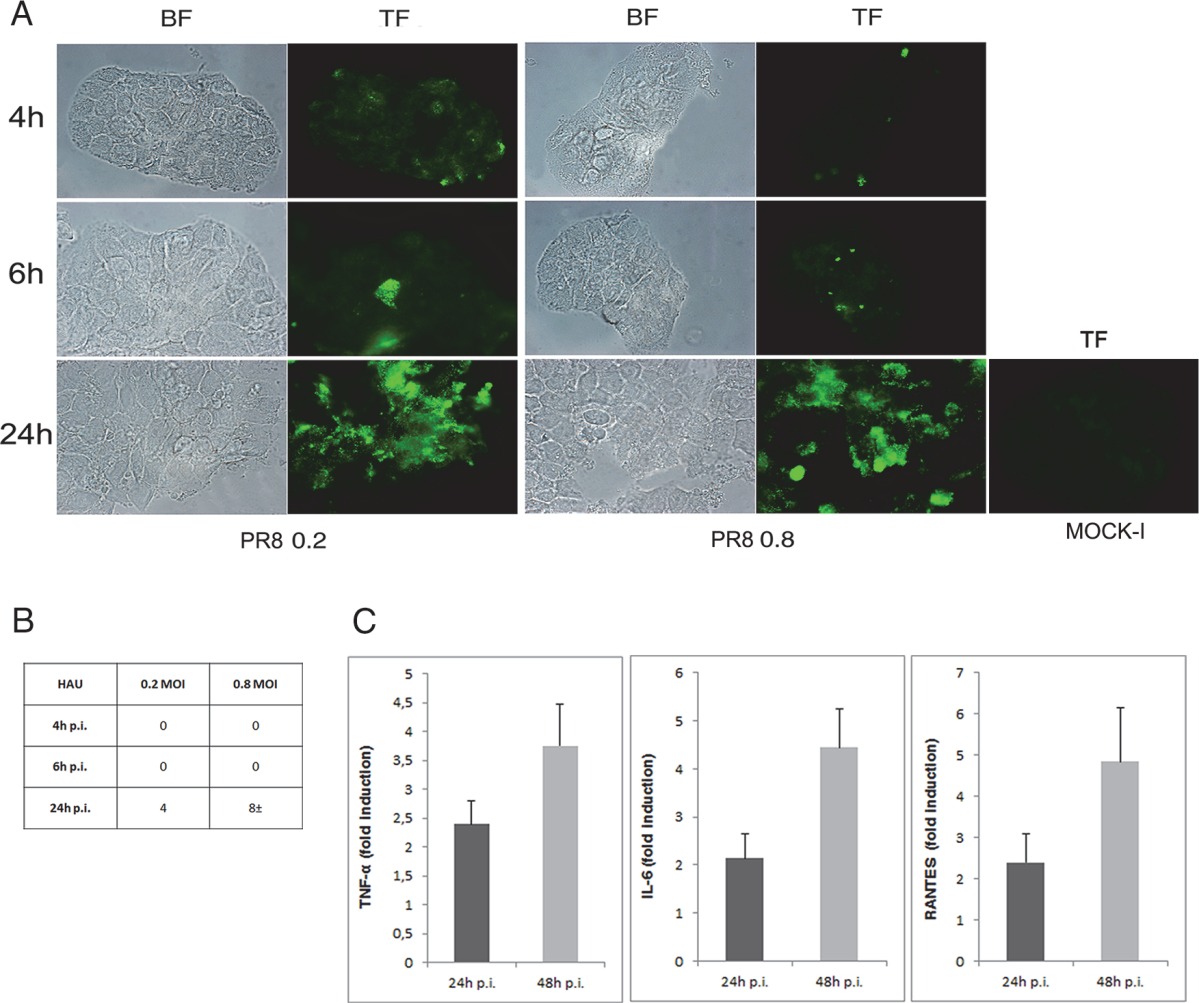
Influenza virus enhances exposure of TF antigen and cytokines secretion in intestinal cells. A) Caco-2 cells were infected with PR8 at 0.2 and 0.8 MOI and maintained for 4, 6 and 24 hrs at 37°C. Cells were fixed and incubated with anti-TF Ab in PR8 infected cells and in the control cells (Mock-I) as described in Methods. Results are shown for one representative experiment of the three performed at magnification of 100X. B) Viral replication was assessed by haemagglutination (HAU/ml) assays in the supernatant of infected cells. C) Cells were infected at 0.8 MOI and supernatants were collected at 24 and 48 hrs p.i. The concentration of pro-inflammatory cytokines (TNF-α, IL-6 and RANTES) was measured by Bioplex multiplex assay. Data are expressed as fold induction of pro-inflammatory cytokines in infected cells relative to control cells. The graphs represent cumulative results of two different experiments.

**Fig 4:**
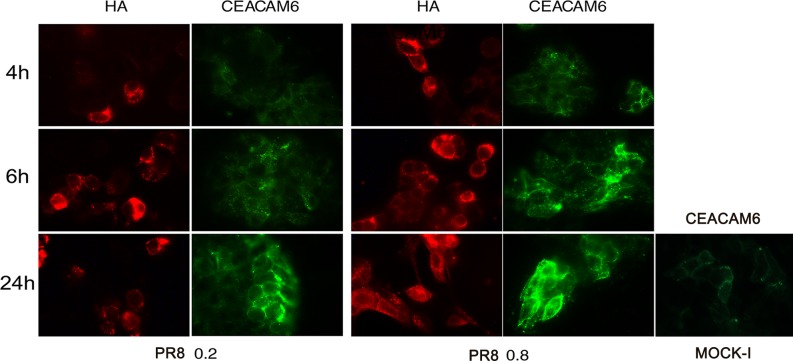
Influenza virus enhances exposure of CEACAM6 receptor in intestinal cells. Caco-2 cells were infected with PR8 at 0.2 and 0.8 MOI and maintained for 4, 6 and 24 hrs at 37°C. Cells were then fixed and samples were incubated with anti-HA to assess viral infection or anti-CEACAM6 Abs to detect antigen expression in PR8 infected cell and in the control cell (Mock-I), (see Methods). BF: Bright field. Results are shown for one representative experiment of the three performed at magnification of 100X.

To confirm that the increase in galactose and mannose residues previously observed were related to TF and CEACAM6 antigens, whose abnormal expression has been documented in CD patients [19–21], we assessed their exposure on Caco-2 cells during IV infection at 48 hrs post plating. Immunofluorescence analysis with specific antibodies was performed in PR8 infected cells for 4, 6 and 24 hrs. The results showed a progressive and time-dependent increase in TF (the percentage of TF positive cells from 4 to 24 hrs p.i ranged from 0.75%±0.01to 53.5%±3 and from 3%±0 to 57%±15, at 0.2 and 0.8 MOI respectively, Fig. 3A) and CEACAM6 exposure (the percentage of positive cells from 4 to 24 hrs ranged from 22.5%±3.5 to 37%±1 at 0.2 MOI and from 28%±11 to 54%±6 at 0.8 MOI, Fig. 4). Viral replication was followed measuring haemagglutinin units (HAU) or labeling the viral glycoprotein HA with specific Abs. As shown in Figs. 3B and 4, virus titer increased during the time of infection and in dose dependent manner. In particular quantification of cells positive to HA labelling, from 4 to 24 hrs p.i., showed an increase in percentage ranged from 11%±1 to 29%±3 at 0.2 MOI and from 24.4%±4 to 46%±9 at 0.8 MOI (Fig. 4). All these results indicated that IV replication led to an over-exposure of both the receptors on the surface of infected cells. In these conditions, the analysis of virus induced inflammatory response showed high production of pro-inflammatory cytokines (TNF-α, IL-6 and RANTES) released in the supernatants of infected cells (Fig. 3C).

**Fig 5:**
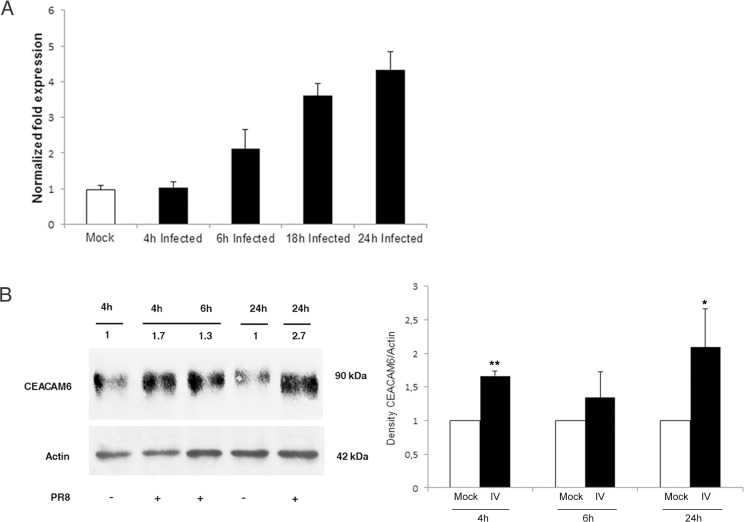
Influenza virus leads to enhanced CEACAM6 mRNA level and protein expression. Caco-2 cells were infected with PR8 (0.8 MOI) and harvested at different times p.i. Samples were used for mRNA or for protein extraction, as described in Methods. A: real-time PCR of CEACAM6 mRNA levels in infected cells harvested at 4, 6, 18 and 24 hrs p.i. Levels of mRNA are expressed as the ratio between CEACAM6 normalized for β-actin and GAPDH. B left panel: a rapresentative western blot analysis of CEACAM6 expression at different times 4, 6 and 24 hrs p.i. As controls, mock-infected cells were recovered at 4 and 24 hrs p.i. Samples were lysed, run onto SDS-PAGE, blotted and immunostained with anti-CEACAM6 Abs. Loading control was Actin. B right panel: densitometric analysis expressed as the ratio of CEACAM6 normalized for actin is reported. The values of western blot signals were obtained by densitometric analysis of control at 4 and 24 hrs respect to infected cell at 4, 6 and 24. Results are shown for the three representative experiments (*P<0.05, **P<0.01).

High levels of CEACAM6 mRNA and protein expression were recently detected in epithelial lung cells infected with IV [25]. Thus the expression of CEACAM6 at transcriptional and translational level was evaluated in our model. Caco-2 cells were infected with PR8 (0.8 MOI), and maintained at 37°C for different times (4, 6, 18, 24 hrs p.i). Cells were detached and used for mRNA and protein extraction, as described in Methods. As shown in Fig. 5A, mRNA levels increased time dependently during PR8 infection, and the ratio of mRNA between IV and mock-infected cells was about 4.5 times higher at 24 hrs p.i. Western blot analysis indicated a similar increase for CEACAM6 protein expression (Fig. 5B, left panel). Densitometric analysis was performed for three different experiments (Fig. 5B, right panel) at 4, 6 and 24 hrs p.i. confirming the increase of the ratio CEACAM6/actin of IV infected cells respect to Mock infected cells.

These results confirmed that influenza A virus led to enhanced CEACAM6 mRNA and protein expression in intestinal epithelial cells.

### Anti-TF and anti-CEACAM6 Abs reduce *E. coli* adhesion on Caco-2 cells pre-infected with influenza virus

To verify whether the increase in bacterial adhesion was due to virus-induced changes in the expression of TF and CEACAM6 receptors, inhibition assays of bacterial adhesion were performed. Caco-2 cells were infected with PR8 for 24 hrs, and incubated at 4°C with anti-TF or with anti-CEACAM6 Abs for the following 2 hrs. The cells were then washed with fresh medium and co-infected with LF82 or S15 *E. coli* strains.

**Fig 6:**
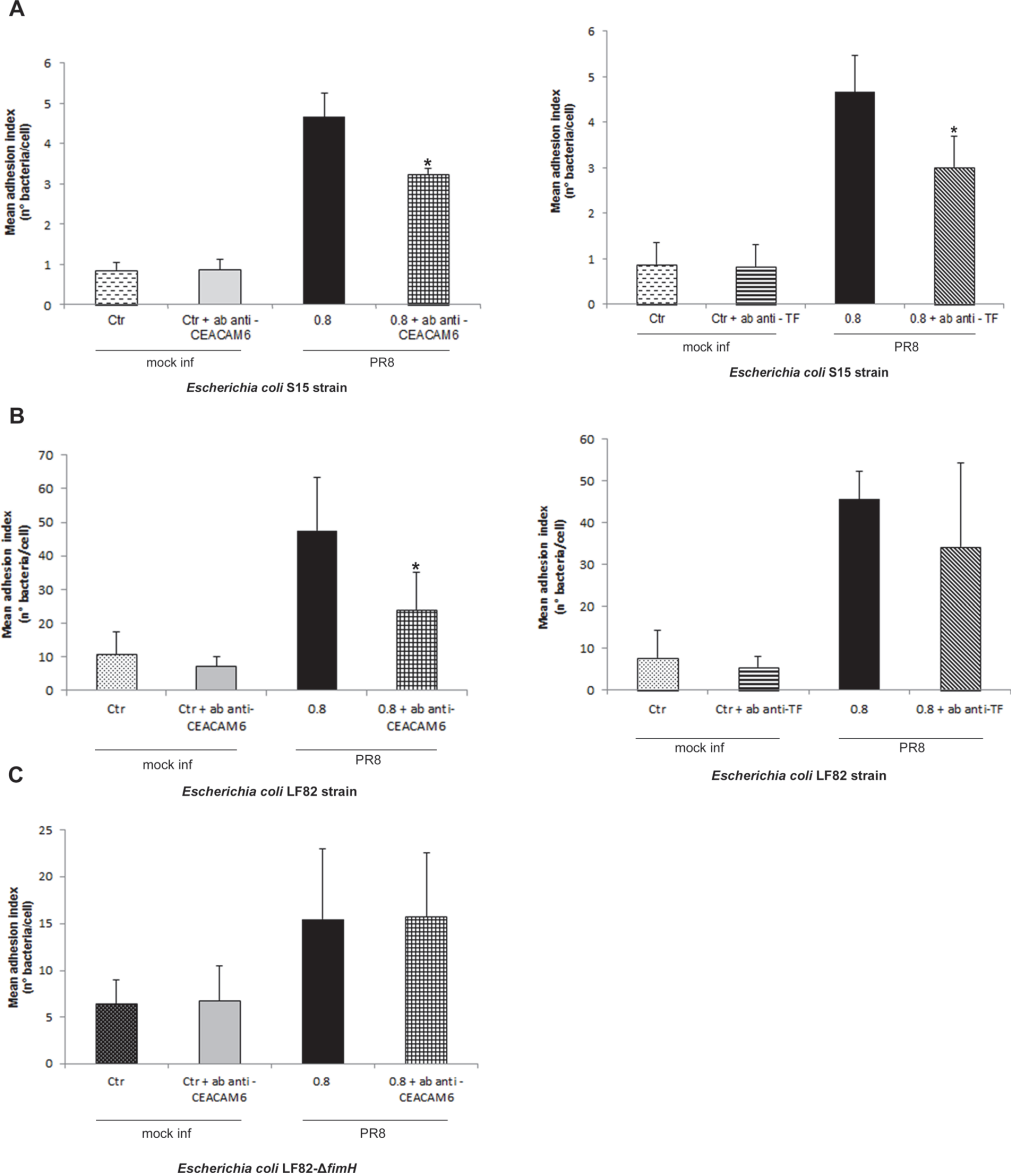
Anti-TF and anti-CEACAM6 Abs reduce bacterial adhesion on PR8-infected cells. Caco-2 cells were infected with PR8 (0.8 MOI) for 24 hrs p.i., and then were incubated with anti-TF or anti-CEACAM6 Abs. After 2 hrs incubation, cells were infected with *E. coli* S15 (A), LF82 (B) and LF82-*ΔfimH* mutant (C) strains, as described in Methods. Data represent the mean ± SD of three independent experiments, each performed in triplicate *P<0.05 vs. untreated infected cells.

Incubation with anti-CEACAM6 Abs caused a significant decrease in the mean level of adhesion to PR8-infected cells of both S15 and LF82 strains. Indeed, the mean adhesion index for S15 strain was 3.25±0.14 in cells treated with anti-CEACAM6 against 4.66±0.58 in untreated cells (Fig. 6A left panel), and for LF82 strain was 24.11±10.92 against 47.58±15.77 in untreated cells (Fig. 6B left panel).

Incubation with anti-TF Abs caused a significant decrease in S15 adhesion level in IV infected cells compared with untreated infected cells: 3.01±0.67 and 4.67±0.8 bacteria per cell, respectively (Fig. 6A right panel). In contrast, the presence of anti-TF Abs did not change the mean adhesion level of *E. coli* LF82 in treated and untreated infected cells (34.38±20.04 and 45.63±6.65, respectively) (Fig. 6B right panel).

To evaluate the involvement of FimH adhesin in bacterial adhesion, PR8 infected cells were incubated with anti-CEACAM6 antibody and co-infected with the LF82 isogenic mutant lacking of *fimh* gene. We found that adhesion index of LF82-Δ*fimH* mutant did not change in presence or absence of CEACAM6 antibody (15.48±7.54 and 15.73±6.80, respectively) (Fig. 6C), suggesting that not only FimH adhesin could be involved in bacterial adhesion during IV infection.

In mock-infected cells, no differences in the adhesion index of the two *E. coli* strains were observed between treated and untreated cells.

Overall the data confirm that the exposure of TF and CEACAM6 antigens observed during IV infection plays a key role in increasing bacterial adhesion to intestinal cells, but we cannot exclude that other cellular receptors could be involved.

### Influenza virus replication leads to inflammation and exposure of TF antigen in primary intestinal epithelial cells

**Fig 7:**
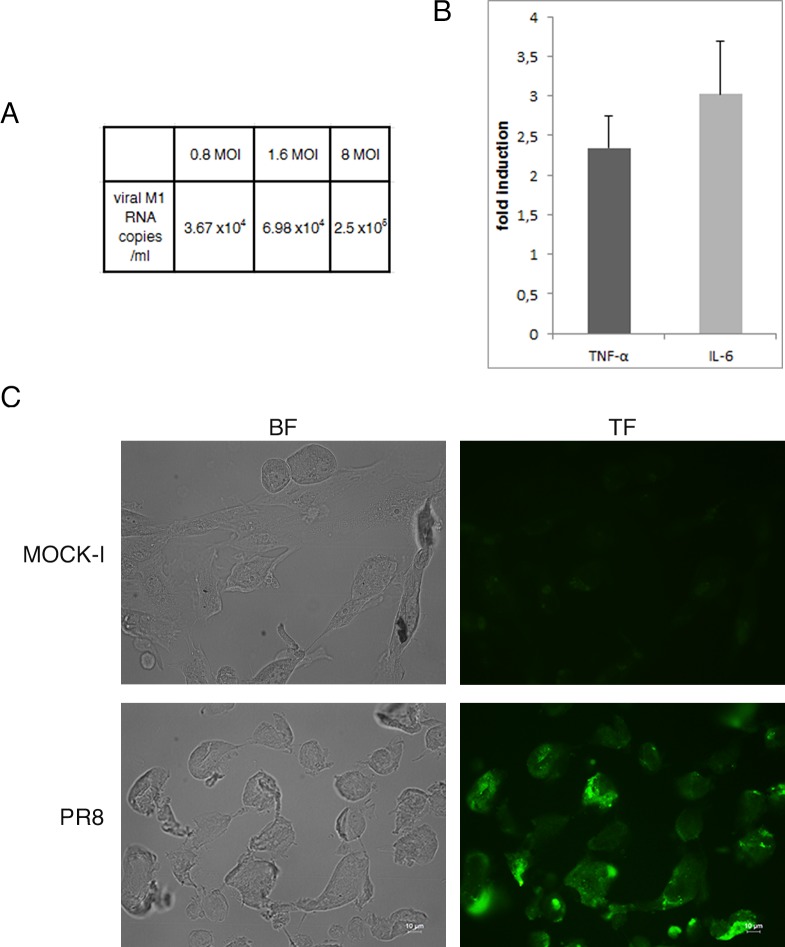
Influenza virus enhances exposure of TF antigen and cytokines secretion in primary cells. A) Viral replication was assessed in the supernatants of intestinal primary infected cells by real time RT-PCR and expressed as number of viral M1 RNA copies/ml. B) Primary intestinal cells were infected with PR8 at 1.6 MOI at 33°C and supernatants were collected at 24 hrs p.i. The concentration of pro-inflammatory cytokines (TNF-α and IL-6) was measured by Bioplex multiplex assay. Data are expressed as fold induction of pro-inflammatory cytokines in infected cells relative to control cells. The graphs represent cumulative results of two different experiments C) Primary were infected with PR8 at 1.6 MOI for 24 hrs at 33°C. Mock infected (MOCK-I) and infected cells were fixed and incubated with anti-TF Ab as described in Methods. Results are shown for one representative experiment of the two performed at magnification of 100X.

TF antigen is over-expressed in colorectal cancer, thus to verify whether IV was able to enhance TF exposure in normal epithelial intestinal cells, viral infection was set up in human primary intestinal epithelial cells using different MOI (range 0.8–8). Viral replication was followed by real time RT-PCR of viral M1 RNA copies/ml in the supernatants of infected cell at 24 hrs p.i. As shown in Fig. 7A viral load increased in dose dependent manner (increase of about one log from 0.8 to 8 MOI). Interestingly, influenza virus was able to induce in these cells the production of pro-inflammatory cytokines (TNF-α and IL-6) (Fig. 7B) as well as the exposure of TF antigen (Fig. 7C). These results indicate that the normal intestinal epithelium may be affected by influenza virus.

## Discussion

In this study, we demonstrate for the first time that influenza A virus significantly increases the adhesive behavior of mucosa-associated *E. coli* strains, inducing the exposure of cellular receptors in intestinal cells.

An increased load of mucosa-associated *E. coli* has been observed in CD patients [17]. Such a phenomenon plays an important role in determining the intestinal dysbiosis typical of these patients in which potentially pathogenic bacteria take over instead of beneficial species. Different factors could favor *E. coli* colonization in CD patients including the specific adhesive behavior related to bacterial genetic features, such as mutations in *fimH* gene and changes in the glycosylation status of specific receptors on the intestinal mucosa [39]. In particular, an over-expression of the mannosylated glycoreceptor CEACAM6 and an increased exposure of the oncofetal TF antigen, have both been reported in CD patients [19,21]. Thomsen-Friedenreich is a cryptic glycoprotein that is absent or masked by carbohydrates in normal colon but exposed in IBD mucosa after the removal of SA from Gal residues. In these conditions, it may be recognized by microbial lectins, thus increasing bacterial adhesion to the GI epithelium [17]. In previous studies, we demonstrated that treatment of Caco-2 cells with purified Cl NA increased the mean adhesion level of *E. coli* isolated from CD patients, suggesting that the over-exposure of TF caused by NA sialidase activity, could be involved in increased bacterial adhesion [18,40]. In the present study, the effectiveness of Cl NA in promoting the adhesion of both the LF82 and the S15 strains was confirmed. We found that IV was more effective in increasing bacterial adhesion compared to treatment with pure Cl NA. Moreover, the enhanced adhesion ability seen in virus-infected cells was related to a progressive increase in TF and CEACAM6 antigen exposition.

By comparing the behavior of the two strains, we found that the clinical isolate S15 was intrinsically less adhesive than the AIEC LF82. This is in line with the genetic features of the two strains. Indeed, LF82 possesses the variant FimH adhesin, which recognizes specifically CEACAM6 [21], while S15 is a *fimh* negative gene strain that, at least theoretically, should not recognize this receptor [18]. Accordingly, the adhesion index of LF82 *ΔfimH* was lower than the index of LF82. However the adhesion ability shown by S15 and LF82 *ΔfimH* to IV infected cells let us to hypothesize that the virus allows binding with other bacterial receptors different from FimH adhesin. It is known for example that long polar fimbriae allow the interaction between AIEC LF82 and Peyer’s Patches [16].

Surprisingly, the increased adhesion of LF82 to virus-infected cells is not completely counteracted by an antibody against CEACAM6, and the antibody against CEACAM6 partially inhibited the adhesion of S15 to IV infected-cells. Moreover in mock-infected cells (Ctr), treated or not with anti-TF or anti-CEACAM6 Abs, we did not observe significant differences in adhesion index for both strains. A possible explanation for this partial inhibition could be an interaction of bacteria with other cellular receptors not yet characterized. As example, over-expression of receptor Gp96, which co-localizes with CEACAM6, has been reported in CD patients, in whom it acts as a receptor for AIEC strains that promote bacterial invasion [41].

Taken together, these results highlight the possibility that more and different cellular receptors, “decrypted” by viral infection, as well as unknown microbial adhesins, could be involved in the bacterial adhesion process.

Host-cell redox changes leading to activation of redox-regulated cellular pathways involved in the control of virus life-cycle have been reported during IV infection [42–45]. Interestingly, the Endoplasmic Reticulum oxidative stress causes the over-expression of the receptor Gp96 observed in CD patients [41]. On the basis of this evidence we can speculate that IV-induced oxidative stress might also contribute to over-expression of some cellular receptors involved in the bacterial adhesion. Further studies are in progress in our laboratory to address this question.

Influenza remains a major cause of morbidity and mortality worldwide that affects large segments of the human population every year [33–46]. Usually IV enters and replicates in cells of the upper respiratory tract where the virus recognizes SA molecules linked to the Gal of glycoprotein on the surface of host epithelial cells [47]. Recently, the same receptors used by the virus have been found abundantly expressed on epithelial cells of the GI tract [26]. Interestingly, Gaur et al. [25] showed that IV infection enhanced survival of lung epithelial cells via interaction of viral NA with CEACAM6, whose expression was significantly increased after viral infection. Accordingly in the present study, we demonstrate that IV is able to enhance CEACAM6 expression in intestinal epithelial cells at both transcriptional and translational level since the early phases of viral infection with a *maximum* at 24 hrs, suggesting that, in our model too, viral NA may act with a similar mechanism.

Over-expression of CEACAM6 in intestinal cells has been reported after stimulation with inflammatory cytokines such as Interferon (IFN)-γ and Tumor Necrosis Factor (TNF)-α, or after infection with *E. coli* AIEC [22]. IV also induces a strong pro-inflammatory response that contributes to immunopathology and clinical symptoms [48]. In the present study we demonstrate that IV induces pro-inflammatory cytokine production in Caco-2 cells and more interestingly in human primary intestinal cells. Although we cannot exclude the possibility that CEACAM6 over-expression in intestinal cells might be related to virus-induced inflammatory pathways, our data suggest that a general perturbation of the cell membrane induced by the virus during its life-cycle may play a pivotal role in unmasking specific surface receptors. TF is an example of antigen that is covered in normal epithelium and is over-expressed in colorectal cancer [49]. We demonstrate that TF is exposed in normal epithelial intestinal cells infected with IV, indicating infection as another condition that can increase TF expression. Further studies will be needed for understanding these mechanisms.

Overall our results demonstrate that IV, by inducing the exposure of cellular receptors, significantly increases the adhesive behavior of mucosa-associated *E. coli* strains potentially involved in the persistence and severity of intestinal inflammation. Thus, IV could constitute an extra risk factor in patients affected by chronic inflammatory disease and we can speculate that vaccination against IV could be useful in individuals genetically susceptible to IBD.
